# Surgical treatment and muscle protein analysis of V-pattern exotropia in craniosynostosis

**DOI:** 10.1038/s41598-022-15707-4

**Published:** 2022-07-07

**Authors:** Qingyu Liu, Yuan Li, Siying Wang, Wenjing Zheng, Han Ye, Wen Li, Tong Qiao

**Affiliations:** Department of Ophthalmology, Shanghai Children’s Hospital, School of Medicine, Shanghai Jiao Tong University, Shanghai, China

**Keywords:** Eye abnormalities, Ocular motility disorders

## Abstract

The purpose of this study was to compare the differences of V-pattern exotropia in craniosynostosis and normal children. 39 children were included in this study, 19 craniosynostosis and 20 children in control group. They underwent comprehensive ocular examinations and received strabismus surgery. The extraocular muscle samples were analysed. Compared with the control group, craniosynostosis group had larger deviation in primary and up gaze, larger V pattern, and more severe inferior oblique overaction. For 20–40, and 50–60 prism diopter exotropia, the lateral recession in the craniosynostosis group was larger than that in the control group, 7.13 ± 0.44 mm vs 6.71 ± 0.47 mm, 8.90 ± 0.21 mm vs 7.75 ± 0.46 mm (*p* = 0.025, 0.000). The anterior transposition of craniosynostosis group was more anterior than that of control group, posterior 1.03 ± 1.24 vs 2.68 ± 0.94 mm (*p* = 0.000). Compared with the control group, the extraocular muscle abnormality in craniosynostosis was significant, 32% vs 5% (*p* = 0.031). There were 40 proteins in craniosynostosis group, which were different from those in control group. A larger V pattern and larger deviation is common in craniosynostosis children. For the same PD of deviation, it usually needs more recession in craniosynostosis because of the thinner and weaker extraocular muscles. Collagen related proteins were increased in craniosynostosis, and decreased contraction related protein tropomodulin might play key role for the weakness of EOMs.

## Introduction

Craniosynostosis is defined as the premature fusion of cranial sutures, leading to restricted growth of the skull, brain, face and central nervous system development^1^. Craniosynostosis occurs in approximately 1 in 2500 children, which are caused by mutations of genes including *FGFR1*, *FGFR2*, *GFGR3*, *TWIST1*, and *EFNB1*. Approximately 85% of cases are isolated and nonsyndromic, involves only one suture. Syndromic craniosynostosis not only affect multiple sutures, but also associated with additional clinical symptoms such as hand and feet, skeletal and cardiac defects, developmental delays. Syndromic craniosynostosis include Crouzon, Apert, Pfeiffer, and Saethre–Chotzen syndromes^2^. The ocular complications of craniosynostosis include eyelid anomalies, ptosis and trichiasis, strabismus, proptosis, corneal surface issues due to exposure, refractive error, and papilledema or optic atrophy related to increased intracranial pressure.

Strabismus is common in craniosynostosis children, with a rate between 39 and 90.9%, compared to 2.1–3.3% in general children^2–4^. Exotropia is more common than esotropia, and V pattern with superior oblique (SO) palsy or inferior oblique (IO) overaction dominates the landscape. Strabismus in children with craniosynostosis is mainly caused by the following two factors^5,6^, abnormal anatomical structure and variation of extraocular muscles. The abnormal facial anatomy results in orbital external rotation and protrusion of eyeball. The medial rectus (MR) moves up and the eyeball turns up during adduction, which is similar to the hyperfunction of IO muscle. Meanwhile the trochlear relative backward, so that the strength of the SO weakened, secondary IO overaction. In addition, the development of extraocular muscles is abnormal, including absent muscles, particularly SO, inferior rectus (IR), and superior oblique (SO). And abnormal insertions, bifid insertions, abnormal orientation, muscle fusion, hypertrophic muscles, and atrophic muscles.

The strabismus and especially the V pattern exotropia are difficult to manage and usually do not respond well to conventional methods^7^. Depending on severity and atypia of the misalignment, remediation can prove challenging. The purpose of this study was to compare the V pattern exotropia between children with craniosynostosis and normal children, to explore the characteristics of strabismus and surgery design, as well as the difference of muscle proteins. Thereby may help to identify appropriate treatment strategies.

## Methods

### Subjects

39 Children were included in this study from May 2017 to October 2020 in Shanghai Children’s Hospital, 19 craniosynostosis children and 20 children in control group. All of the children were diagnosed with V pattern strabismus and received surgery. They underwent comprehensive ocular examinations before surgery. We recorded preoperative characteristics including age, sex, duration of strabismus, visual acuity, diopter, prism diopters. The presence of a V pattern > 15∆ was clinically observed and was quantified by measuring the deviation by the prism and alternate cover test with the eyes in a position of 25° upgaze and 25° downgaze. Versions were graded on a scale of − 4 underaction to + 4 overaction, with 0 being normal. This study’s protocol adhered to the Declaration of Helsinki and was approved by the Institution Review Board of Shanghai Children’s Hospital (2020R122-E01), The clinical registration number was ChiCTR2000038770. Written informed consent was obtained from each patient’s parent before surgery.

### Surgery procedure

All surgeries were performed under general anesthesia. The operations were performed according to the surgical protocol modified from the surgical formula proposed by Wright^8,9^. Horizontal rectus muscle surgery was performed to correct the horizontal deviation, including bilateral or unilateral lateral rectus recession, or recession-resection (lateral rectus recession and medial rectus resection).

The amount of anterior transposition of IO was determined by both the severity of the IO overaction and the extent of the V pattern^10^, moving the anterior fibers of the IO muscle insertion to a location along 2 mm temporal border of the IR muscle (0 mm, 1 mm, 2 mm, 3 mm, 4 mm posterior). The IO muscles of patients with asymmetric IO overaction were reattached asymmetrically. No adjustable sutures were used. Resected tissue fragments of the extraocular muscles were obtained from patients during strabismus surgery at Shanghai Children’s Hospital. The tissue samples were frozen in liquid nitrogen at − 80 °C after resection and stored until use.

### Relative quantitative analysis of label free proteome by nanoliter liquid chromatography quadrupole orbitrap trap mass spectrometer

Samples were add to pyrolysis solution. After centrifugation at 12,000 RPM for 30 min at 4 °C, the supernatant is transferred to a new centrifuge tube. Protein concentration was measured using a bicinchoninic acid assay (BCA) protein assay kit (Pierce, Rockford, IL). Then, proteins were quantitatively taken and supplemented with NH4HCO3 to 100 μl, incubated with 1 M Dithiothreitol solution for 1 h at 37 °C. 1 M iodoacetamide solution was added and kept away from light for 40 min at room temperature. After centrifugation, the precipitations were digested with trypsin (w (trypsin):w (protein) = 1:25) at 37 °C overnight. After centrifugation, the enzymatic hydrolysis products were redissolved with 20 μl 0.1% formic acid solution, desalted with C18 micro column and dried. 1% formic acid solution was used to re dissolve the solution.

C18 reversed phase column (75 μm × 20 cm, 3 μm) was used for the analysis of nanoliter liquid chromatography. The mobile phase a was a mixture of 99.9% water and 0.1% formic acid, and the mobile phase B was a mixture of 80% acetonitrile and 0.1% formic acid. The liquid gradient was 0–3 min, 2% B; 3–95 min, 6–20% B; 95–107 min, 20–32% B; 107–108 min, 32–100% B; 108–120 min, 100% B. The flow rate of mobile phase was 500 nl/min. In ESI + mode, full scan acquisition (m/z 350–1800) was performed in a 70 000 resolution (AGC 3e6) orbital well. The first 20 peptide signals (excluding the parent ions with charge 1, 7, 8 and greater than 8) were broken by high energy collision (HCD), and the normalized collision energy (NCE) was 28.0. The capillary temperature is 275 °C and the spray voltage is 2000 V. The sub ions were measured at a resolution of 17,500 (AGC 1E5). The maximum fill time of full scan and MS–MS scan were set to 50 ms and 45 ms respectively, and the dynamic exclusion time was set to 30 s.

### Statistical analyses

Statistical analyses were performed using SPSS software version 24 (IBM-SPSS, Chicago, IL, USA). Descriptive statistics are reported herein as mean ± standard deviations (SD). Statistical comparisons were performed using *t* test. A *p* value of < 0.05 was considered statistically significant.

Protein identification and quantification of the data were performed using Protein Discoverer 2.4 (Thermo Fisher Scientific). The proteomic database used in this study was the Homo sapiens (sequence 20148 http://www.uniprot.org/uniprot). Search database parameter setting as following, fixed modification: carbamodomethyl (c). Variable modification: oxidation (m), deamidation (n, q), acetyl (N-terminus). Precursor mass tolerance 10 ppm, fragment mass tolerances 0.02 da, and max missed cleavages 2. The differential expressed proteins were set: ≥ 2 was up-regulated (*p* < 0.05) and ≤ 0.8 was down-regulated (*p* < 0.05).

## Results

A total of 39 patients diagnosed with V pattern strabismus were included in this study, 19 craniosynostosis children and 20 children in control group. There were 13 males and 6 females in craniosynostosis, 12 males and 8 females in control group. Average age of craniosynostosis was 4.37 ± 1.74 (range 2–8 years), while control group was 4.85 ± 1.60 (range 2–7 years). Craniosynostosis types were 14 Crouzon syndrome, 1 Pfeiffer, and 3 UCS (unilateral coronal synostosis). There were 8 cases with concomitant ophthalmic diseases in craniosynostosis group, including ptosis, entropion, and nystagmus. A summary of the preoperative patient characteristics is shown in Table 1. Duration of strabismus was longer in craniosynostosis than control group, which indicating an earlier onset age (*p* = 0.011). Compared with the control group, craniosynostosis group had larger deviation in primary and up gaze, larger V pattern, and more severe IO overaction (*p* = 0.049, 0.002, 0.000, 0.001). There was no difference in gender distribution and incidence of amblyopia.

**Table 1:** Preoperative characteristics of the studied patients.

	Craniosynostosis (n = 19)	Control group (n = 20)	*p*
Age at surgery (years)	4.37 ± 1.74	4.85 ± 1.60	0.373^a^
Sex (male:female)	13:6	12:8	0.584^b^
Duration of strabismus (years)	3.53 ± 1.84	2.00 ± 1.71	0.011^a^
Amblyopia (n, %)	6, 32%	3, 15%	0.219^b^
Deviation in primary position (PD)	52.37 ± 22.94	41.50 ± 6.71	0.049^a^
Deviation in up gaze (PD)	81.05 ± 34.14	55.00 ± 10.51	0.002^a^
Deviation in down gaze (PD)	32.11 ± 23.47	31.75 ± 6.74	0.949^a^
V pattern (PD)	48.95 ± 18.83	23.25 ± 6.13	0.000^a^
IO overaction (+ 1 to + 4)	2.71 ± 0.98	1.98 ± 0.80	0.001^a^
SO underaction (− 1 to − 4)	− 1.13 ± 1.40	− 0.28 ± 0.60	0.001^a^
Extraocular muscle abnormality	6, 32%	1, 5%	0.031^b^

^a^p value by the independent *t* test, ^b^p value by the Chi-square test.

*PD* prism diopter, *IO* inferior oblique, *SO* superior oblique.

### Surgical comparison

The surgery design was shown in Table 2. Most of the operations used bilateral lateral rectus recession (BLR), and some used unilateral lateral rectus recession (ULR) or combined with medial rectus resection (MR). After grouping and comparing different levels of deviation, it was found that for 20–40, and 50–60 PD exotropia, the lateral recession in the craniosynostosis group was larger than that in the control group, 7.13 ± 0.44 mm vs 6.71 ± 0.47 mm, 8.90 ± 0.21 mm vs 7.75 ± 0.46 mm (*p* = 0.025, 0.000). The same situation was found in 50 PD deviation, 8.83 ± 0.29 mm vs 7.50 ± 0.00 mm (p = 0.015). There was a range of 15–90 PD of exotropia in the craniosynostosis group, compared with 30–60 PD in the control group.

**Table 2:** Comparison of the operation between craniosynostosis and control group.

		n	Craniosynostosis (mm)	n	Control group (mm)	p
Horizontal surgery		19	7.16 ± 2.65	20	6.93 ± 0.63	0.704
	20–40∆	8 eyes	7.13 ± 0.44	32 eyes	6.71 ± 0.47	0.025
	50–60∆	10 eyes	8.90 ± 0.21	8 eyes	7.75 ± 0.46	0.000
	15∆	2	0	0		
	20∆	1	ULR 7.5	0		
	30∆	1	ULR 7.5	2	6.75 ± 1.96	
	40∆	3	7.00 ± 0.50	14	6.71 ± 0.43	0.320
	50∆	3	8.83 ± 0.29	3	7.50 ± 0.00	0.015
		1	ULR 8 MR 6			
	60∆	2	9.00 ± 0.00	1	8.5	
		1	BLR 7 MR 4			
	80∆	4	BLR 7.83 ± 0.76 MR 4.83 ± 0.76	0		
	90∆	1	BLR 9 MR 6	0		
IO posterior		38 eyes	1.03 ± 1.24	40 eyes	2.68 ± 0.94	0.000
	0–2+	15 eyes	1.67 ± 1.45	31 eyes	3.06 ± 0.57	0.002
	3–4+	23 eyes	0.61 ± 0.89	9 eyes	1.33 ± 0.71	0.037

*ULR* unilateral lateral recession, *BLR* bilateral lateral rectus recession, *MR* medium rectus resection.

In terms of IO operation, the anterior transposition of craniosynostosis group was more anterior than that of control group, posterior 1.03 ± 1.24 vs 2.68 ± 0.94 mm (*p* = 0.000). According to the degree of IO overaction, they were divided into 0–2+, 3–4+, and the contrast was still the same (1.67 ± 1.45 vs 3.06 ± 0.57 mm, *p* = 0.002, 0.61 ± 0.89 vs 1.33 ± 0.71 mm, *p* = 0.037).

**Figure 1:**
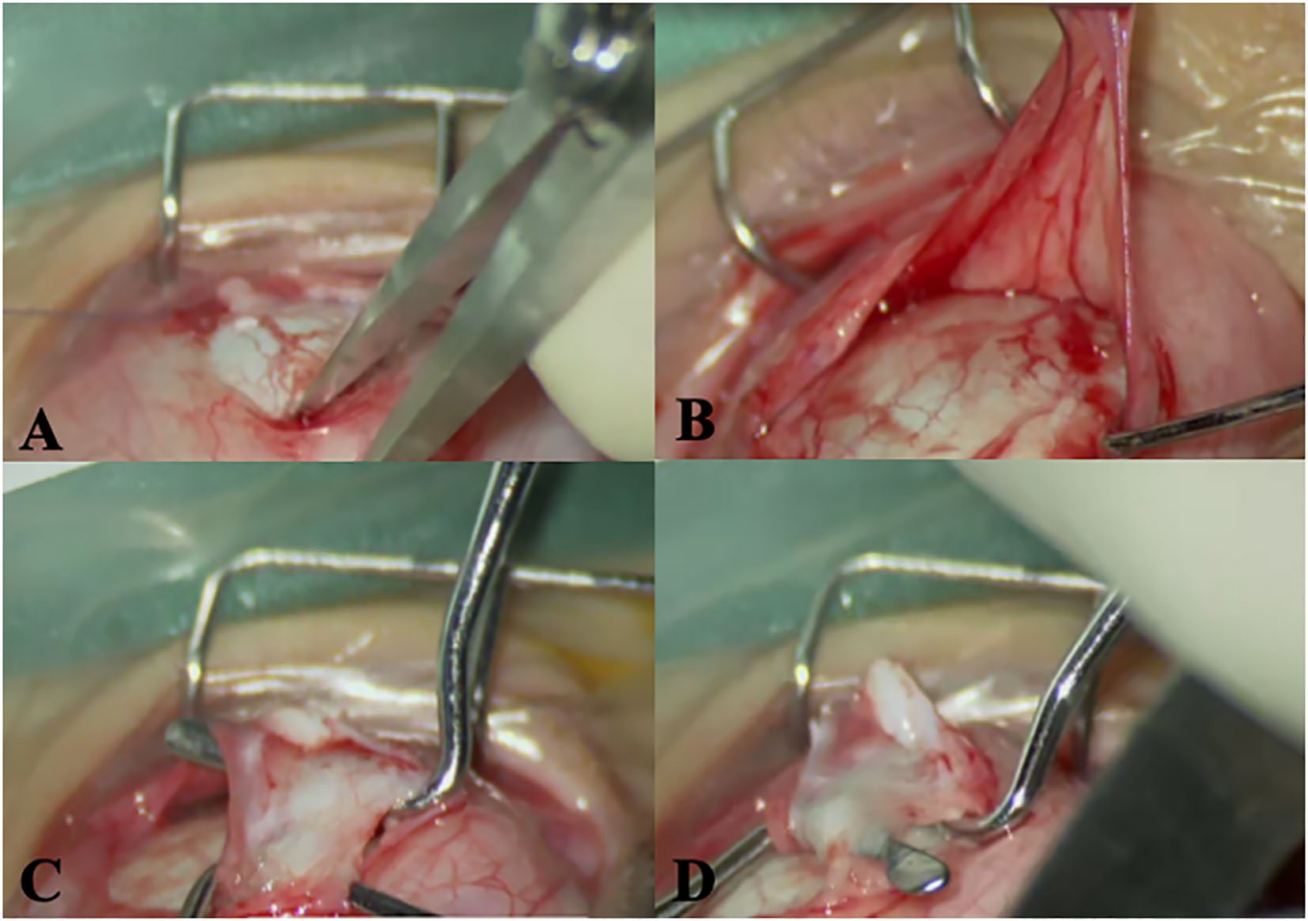
Extraocular muscle abnormalities of craniosynostosis. (**a**,**b**) IR absence, (**c**) LR wedge-shaped deformity, (**d**) LR ectopic and adhesion to the lower sclera.

There were 6 cases of extraocular muscle abnormalities in craniosynostosis group, including 5 IR absence, with or without lateral rectus ectopic, wedge-shaped deformity, adhesion to the lower sclera, and IO dysmorphia (Fig. 1). There was 1 case with IO muscle two bundles in the control group. Compared with the control group, the extraocular muscle abnormality in craniosynostosis was significant, 32% vs 5% (*p* = 0.031). The operation plan was adjusted after the unpredictable muscle abnormality was found during the surgery. The IO muscle anterior transposition was suture to the temporal side 6.5 mm inferior the limbal, where supposed to be the IR muscle attachment.

### Proteomics of ocular muscles

**Figure 2:**
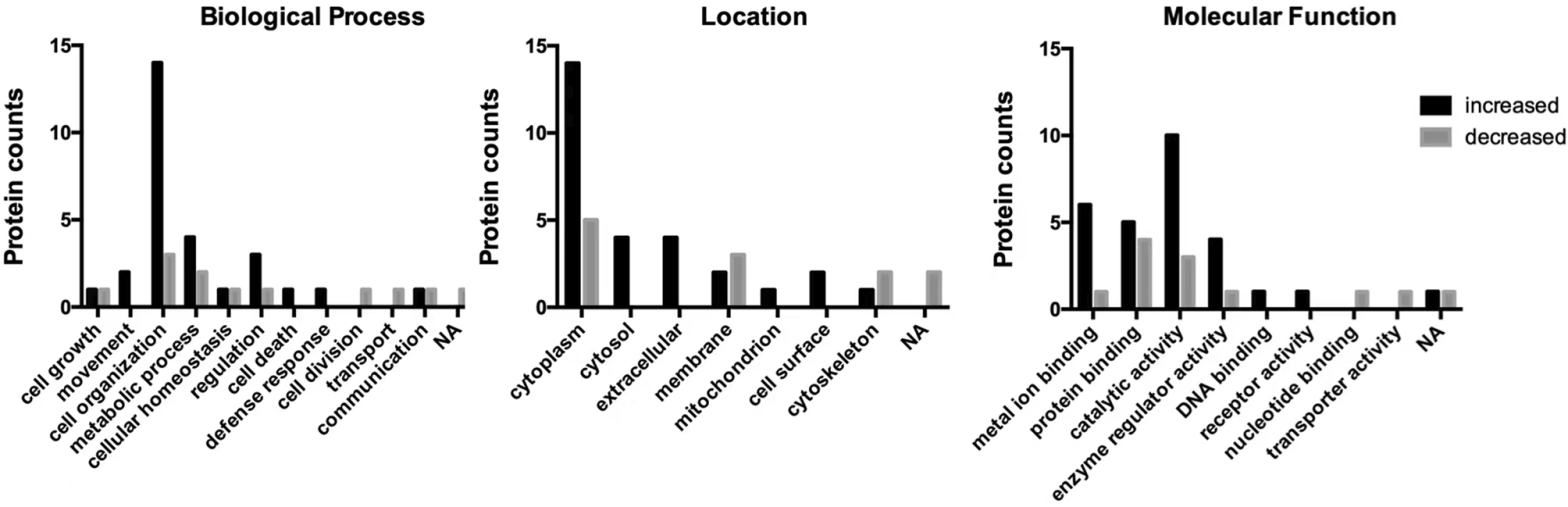
The characteristics of different extraocular muscles proteins in craniosynostosis group.

A total of 2593 proteins were successfully identified. There were 40 proteins in craniosynostosis group, which were different from those in control group (Table 3). To characterize the molecular features, the biological process, location, and molecular function were investigated (Fig. 2). 28 proteins were increased in craniosynostosis group, biological process including cell organization (50%), metabolic process (14%), and regulation (11%). Cell location including cytoplasm (50%), cytosol (14%), and extracellular (14%). Molecular function including catalytic activity (36%), metal ion binding (21%), and protein binding (18%). 12 proteins were decreased, including cell organization (25%) and metabolic process (17%). Cell location including cytoplasm (42%) and membrane (25%). Molecular function including protein binding (33%) and catalytic activity (25%). The altered proteins were classified by functional pathways, including muscle contraction related proteins, collagen related proteins, proteoglycans, and other proteins (Table 4). In craniosynostosis samples, collagen related proteins were increased.

**Table 3:** Changed proteins of extraocular muscle in craniosynostosis versus control.

Accession	Name	Biological process	Location	Molecular function	Ratio
Q96HC4	PDZ and LIM domain protein 5	Cell growth	Cytoplasm	Metal ion binding	100.00
P02585	Troponin C	Movement	Cytosol	Metal ion binding	100.00
P29972	Aquaporin-1	Cell organization	Cytoplasm	Protein binding	36.56
P04430	Immunoglobulin kappa variable 1–16	Movement	Extracellular	Catalytic activity	33.14
Q9NX14	NADH dehydrogenase	Cell organization	Membrane	Protein binding	21.85
P55290	Cadherin-13	Cell organization	Cytoplasm	Metal ion binding	16.35
P35609	Alpha-actinin-2	Cell organization	Cytoplasm	Metal ion binding	13.67
Q6NVY1	3-Hydroxyisobutyryl-CoA hydrolase	Metabolic process	Mitochondrion	Catalytic activity	11.14
P78539	Sushi repeat-containing protein SRPX	Cell organization	Cell surface	NA	6.46
Q16363	Laminin subunit alpha-4	Cell organization	Cytoskeleton	Catalytic activity	4.91
P39023	60S ribosomal protein L3	Cell organization	Cytoplasm	Protein binding	4.56
Q96FJ2	Dynein light chain 2	Communication	Cytoplasm	Catalytic activity	4.52
Q07960	Rho GTPase-activating protein 1	Regulation	Cytoplasm	Enzyme regulator activity	3.94
P49247	Ribose-5-phosphate isomerase	Metabolic process	Cytosol	Catalytic activity	3.40
Q9UH65	Switch-associated protein 70	Cell organization	Cytoplasm	DNA binding	3.30
P38606	V-type proton ATPase catalytic subunit A	Cellular homeostasis	Cytosol	Catalytic activity	3.18
P24844	Myosin regulatory light polypeptide 9	Regulation	Cytosol	Metal ion binding	3.02
Q15149	Plectin	Cell organization	Cytoplasm	Protein binding	2.92
O43866	CD5 antigen-like	Cell death	Cytoplasm	Receptor activity	2.69
P07585	Decorin	Cell organization	Cytoplasm	Enzyme regulator activity	2.57
P52907	F-actin-capping protein subunit alpha-1	Cell organization	Cytoplasm	Protein binding	2.56
Q2UY09	Collagen alpha-1(XXVIII) chain	Regulation	Extracellular	Enzyme regulator activity	2.52
Q9UHG3	Prenylcysteine oxidase 1	Metabolic process	Membrane	Catalytic activity	2.38
P00736	Complement C1r subcomponent	Defense response	Extracellular	Catalytic activity	2.36
P14543	Nidogen-1	Cell organization	Cell surface	Metal ion binding	2.21
Q15056	Eukaryotic translation initiation factor 4H	Cell organization	Cytoplasm	Catalytic activity	2.14
Q13885	Tubulin beta-2A chain	Cell organization	Cytoplasm	Catalytic activity	2.10
P19823	Inter-alpha-trypsin inhibitor heavy chain H2	Metabolic process	Extracellular	Enzyme regulator activity	2.05
P61160	Actin-related protein 2	Cell division	Cytoplasm	Nucleotide binding	0.46
P02730	Band 3 anion transport protein	Cellular homeostasis	Cytoskeleton	Protein binding	0.45
P18577	Blood group Rh(CE) polypeptide	Transport	Membrane	Transporter activity	0.38
P17980	26S protease regulatory subunit 6A	Metabolic process	Cytoplasm	Catalytic activity	0.37
P29274	Adenosine receptor A2a	Communication	Membrane	Catalytic activity	0.36
Q9NVH1	DnaJ homolog subfamily C member 11	Cell organization	Membrane	Protein binding	0.31
P02008	Hemoglobin subunit zeta	Regulation	NA	Metal ion binding	0.28
Q8IY85	EF-hand calcium-binding domain-containing protein 13	NA	NA	NA	0.23
P46940	Ras GTPase-activating-like protein IQGAP1	Cell growth	Cytoplasm	Enzyme regulator activity	0.18
Q15631	Translin	Metabolic process	Cytoplasm	Catalytic activity	0.17
Q14254	Flotillin-2	Cell organization	Cytoskeleton	Protein binding	0.15
Q9NYL9	Tropomodulin-3	Cell organization	Cytoplasm	Protein binding	0.10

*NA* no annotation.

**Table 4:** Changed proteins classified by pathway.

Pathways	Increased	Decreased
Contraction related proteins	Troponin C, Immunoglobulin kappa variable 1–16, Alpha-actinin-2, Myosin regulatory light polypeptide 9	Actin-related protein 2, Adenosine receptor A2a, Tropomodulin-3
Collagen related proteins	Plectin, Collagen alpha-1(XXVIII) chain	
Proteoglycans	Laminin subunit alpha-4, Decorin	Ras GTPase-activating-like protein IQGAP1
Other proteins	PDZ and LIM domain protein 5, Aquaporin-1, NADH dehydrogenase, Cadherin-13, 3-hydroxyisobutyryl-CoA hydrolase, Sushi repeat-containing protein SRPX, 60S ribosomal protein L3, Dynein light chain 2, Rho GTPase-activating protein 1, Ribose-5-phosphate isomerase, Switch-associated protein 70, V-type proton ATPase catalytic subunit A, CD5 antigen-like, F-actin-capping protein subunit alpha-1, Nidogen-1, Prenylcysteine oxidase 1, Complement C1r subcomponent, Eukaryotic translation initiation factor 4H, Tubulin beta-2A chain, Inter-alpha-trypsin inhibitor heavy chain H2	Band 3 anion transport protein, Blood group Rh(CE) polypeptide, 26S protease regulatory subunit 6A, DnaJ homolog subfamily C member 11, Hemoglobin subunit zeta, EF-hand calcium-binding domain-containing protein 13, Translin, Flotillin-2, D

## Discussion

V-pattern exotropia is defined as 15 PD or greater exotropia in up gaze than in down gaze, which is usually associated with IO overaction. IO muscle weakening is used to improve the pattern^11^. V-pattern exotropia strabismus is common in craniosynostosis children, with as many as two thirds of patients manifesting the condition. Anatomic changes to the orbit in craniosynostosis have been postulated to result in relative sagittalization of the origin of the IO, causing the application of Hering’s law to extraocular muscles that are anatomically excyclorotated in craniosynostosis patients^7,12^. Another theory considered that IO overaction results from enhanced contact of the IO with the floor of the globe. A number of publications report on the absence of muscles and attribute to the atypical patterns of strabismus. Craniosynostosis is a complex disorder producing complicated strabismus, which is very difficult to correct surgically.

We found that there was no significant difference in the age of strabismus operation between children with craniosynostosis and normal children, but the duration of strabismus was longer, suggesting that the age of strabismus onset was earlier. There is no definite conclusion about the sequence of strabismus surgery and craniofacial surgery in craniosynostosis children^2^, but cranioplasty surgery must be performed first to relieve intracranial hypertension.

Comparing with the control group, the deviation in primary and up gaze was larger. A larger V pattern was significant in craniosynostosis, which was consistent with the IO overaction and SO underaction. We proposed that these were the characteristic of V-pattern strabismus in craniosynostosis, a larger V sign and large deviation due to abnormal orbital anatomical structure.

Different surgical techniques have been used on strabismus in craniosynostosis. Several studies have examined the best way to correct the V-pattern and over-elevation in adduction^7,13–15^. This problem is complex and difficult to cure with surgery. Denervation/extirpation and myectomy of the IO muscle offered modest benefits, though neither procedure resulted in normalization of ocular motility. Transposition of the rectus muscles in combination with weakening of the oblique muscles is effective. SO tuck may also provide effective reversal excyclotorsion.

In this study, we used the IO muscle anterior transportation combined with horizontal strabismus surgery to correct the V pattern. For the same deviation, the lateral recession was larger in craniosynostosis than that in the control group, suggesting muscles were weaker in craniosynostosis. In the IO muscle anterior transportation, the transportation in craniosynostosis was more anterior than control group. During the surgery, it was difficult to hook the IO muscle, because the position was lower because of the narrow orbit and proptosis in craniosynostosis. We found that the extraocular muscles were thinner and weaker in craniosynostosis children, and the IO muscle was surrounded by fat tissues. Extraocular muscle abnormalities was more common in craniosynostosis children.

In the past decades, significant progress has been made in understanding the genetic basis of craniosynostosis with mutations in the fibroblast growth factor (FGF) signaling pathway. They are important in neuronal differentiation, angiogenesis, wound healing, limb development, and mesoderm induction^16,17^. In strabismus patients, the molecular composition of extraocular muscles was altered, including myosins, tropomyosins, troponins, and collagen related proteins^18^. However, there are few studies on the molecular and protein difference between craniosynostosis and common strabismus extraocular muscles.

We used to report the management of V-pattern strabismus with marked inferior rectus loss in craniosynostosis, and there were pathological changes and collagen degeneration in extraocular muscles^19^. In this study, proteomic analysis of extraocular muscles was performed. According to the classification of different proteins in extraocular muscles, we found that the increased protein functions including catalytic activity, metal ion binding, and protein binding. The decreased protein functions including protein binding and catalytic activity. The altered proteins were classified by functional pathways, including muscle contraction related proteins, collagen related proteins, proteoglycans, and other proteins. Collagens are the main constituents of tendon^20^. Proteoglycans space and lubricate tendons and contribute to fibril fusion and myogenesis^21^. Collagens were found increased in strabismic EOM samples^18^. Compared with common strabismus, the collagen related proteins in EOMs of craniosynostosis were increased, which is consistent with muscle abnormalities and collagen degeneration.

However, we found that there were both increased and decreased proteins in contraction related proteins and proteoglycans in craniosynostosis, which did not seem to explain the weakening strength of EOMs. Meanwhile, our study found that troponin C, myosin regulatory light polypeptide 9, and collagen alpha-1 chain were higher in the extraocular muscles of craniosynostosis group than in the control group. Troponin was contraction-related proteins. Myosins were instrumental in regulating contraction force and velocity of muscle fibers^22^. However, they were reported downregulated in strabismic muscles^18^. We speculate this is related to the abnormal development of craniosynostosis. In craniosynostosis, FGFR mutations are likely to cause ligand independent activation of the receptor, leading to an upregulation of signaling pathways, mutations in the basic helix–loop–helix transcription factor twist appear to induce loss of protein function^16^. The cells derived from muscle were significant more osteogenic and higher alkaline phosphatase expression in craniosynostosis rabbit than wild type^23^.

In craniosynostosis samples, tropomodulin (Tmod) was decreased significantly. Tmod family of proteins are dynamic caps that inhibit actin monomer association and dissociation from actin filament pointed ends. They also regulate the tightness of actin filament pointed-end capping and actin filament stability and lengths. It was reported that reduced Tmod levels or functional deficits could be associated with hereditary myopathies in humans^24^. Tmod deletion in mice produces mild muscle pathology with depressed isometric stress production^25^. Its activity is required for maintenance of a functional contractile apparatus. A Tmod homological protein was expressed in slow fibers of EOMs and might be involved in thyroid-associated ophthalmopathy^26^. Tmod decreased significantly in the EOMs in craniosynostosis children, which might play key role for the weakness of muscle strength.

We compared the differences of V-pattern exotropia, surgery characteristics, and muscle proteins between craniosynostosis group and the control group. A larger V pattern and larger deviation is common in craniosynostosis children. For the same PD of deviation, it usually needs larger recession in craniosynostosis because of the thinner and weaker extraocular muscles. And extraocular muscle abnormalities was more common in craniosynostosis children.

The limitation is that more sample and follow-up are needed. We will continue to study in our future work.

## Conclusions

A larger V pattern and larger deviation is common in craniosynostosis children. For the same PD of deviation, it usually needs more recession in craniosynostosis because of the thinner and weaker extraocular muscles. And extraocular muscle abnormalities was more common in craniosynostosis children. Collagen related proteins were increased in craniosynostosis, and decreased contraction related protein Tmod might play key role for the weakness of EOMs.

## Data Availability

The data is available. The principal investigator and corresponding author have full access to all the data in the study and take responsibility for the integrity and the accuracy of the data analysis.
